# Editorial Department

**Published:** 1881-04

**Authors:** 


### The Bistoury.

ELMIRA, N. Y., APRIL, 1881.

The BISTOURY is published Quarterly, upon the first of April, July, October and January, at Fifty Cents a year in advance.

### Editorial Department.

NEW TYPE.—This number of the BISTOURY is printed with new type.

THE ARRANGEMENT.—Dick arranges these editorial squibs of ours according to their size rather than their value. We sincerely hope that none of the small ones will "get away."

BEN H. PRATT IN WASHINGTON.—It will be seen from Mr. Pratt's contribution in this number that he has been to the inauguration to be squeezed, coddled and bamboozled. But read what he has to say about it and be entertained.

PREMIUM LIST.—We call attention to the premiums offered those soliciting subscribers to the BISTOURY. Our cash premiums are particularly liberal. We will be glad to have any of our subscribers work for us to increase the circulation of our journal.

BODINES.—We learn that the dam on the Lycoming at "Bodines," where every June we have been accustomed to camp, has been swept away by the spring freshet. But then, Robt. Innis promises to have it in place again by camping time, and he always keeps his word.

CHANGE IN EDITORIAL HEADINGS.—With the new volume and new type we make also a change in the editorial department. Hereafter, whatever the editor may have to present, be the same in long or short articles, will come under the one general head of Editorial Department.

ERROR IN TITLE PAGE.—We call attention to an error on the title page sent out with the index, in our last number. It should read from April, 1878, to April, 1881. One entire year would be excluded from the binding by including only the years called for on the title page. Strike out the 9 and substitute an 8, before it is forgotten.

THE NEW PRESIDENT.—President Garfield commences his administration under the most favorable auspices, having secured the respect and confidence of both political parties, through his patriotic utterances and from his evident endeavor to do the very best he can for the whole country. He seems to be a wise and capable executive. Let us all applaud him as his acts may warrant.

OUR SUNDAY PAPERS.—Before a fellow had a chance to make a complimentary notice of the *Sunday Republican*, behold it has ceased to exist! Like a mother's darling—it was too good to live, so it died. The venture seems to have established the fact that there was no need for another Sunday paper in this city, that field being ably and fully filled by the sprightly and enterprising *Telegram*.

ELMIRA COLLEGE.—This excellent institution of learning, especially designed for the education of girls, is one of the very best in our country. A new wing has just been finished and will be occupied within the month, making the already commodious building still more convenient for the large number of pupils in attendance. The faculty is an unusually capable one and the curriculum of studies equal to any similar institution in the land. An effort is now being make to endow it still more handsomely. We hope it will succeed.

SCHWAB.—How about that New York idiot, Schwab? He wants to murder every ruler of every land and every man who has accumulated more property than himself. As Schwab is a trafficker in lager beer, it would be interesting to know just how much property he has himself acquired. Such fellows usually commence building fine blocks of houses after having been in "business" a very few years. Let the "Socialists" therefore interview Schwab, and learn whether it isn't about time to strangle him.

GENERAL HANCOCK.—This gentleman and soldier has acquitted himself with becoming dignity and amiability throughout the presidential election and latterly at the inaugural ceremonies of the gentleman who defeated him. It was a gracious act upon the part of the defeated candidate to lend his presence in Washington upon invitation of the committee having the inaugural ceremonies in hand. It is by acts of this character that the animosities and bitter feelings engendered during a heated campaign are expunged, and a Nation once more restored to equanimity of temper. General Hancock deserves praise from the American people for the part he played at the inaugural ceremonies.

SURGEON GENERAL.—It is expected that Surgeon General Barnes of the U. S. Army will be retired, he having reached an age at which that course is usually pursued. The press, the people, and the medical men of the army in particular, are asking for the appointment of Surgeon J. H. Baxter, chief medical Purveyor of the Army. Dr. Baxter is a gentleman qualified professionally, socially and in every other particular, for the position, and no appointment so far made by the President, will be more universally approved than that of Dr. Baxter to the Surgeon Generalship.

SPECIMEN COPIES.—We have our opinion (reserved) of those good but thoughtless people who order "sample copies" of the BISTOURY by postal card. If we were only now and then called upon to fill such orders, we might easily comply by sending the journal and paying postage thereon, gratuitously, just to afford many contemptibly mean people free literature. But such orders are altogether too frequent. It becomes burdensome to pay postage even, to say nothing of the cost of the journal. Hereafter, no "specimen copies" will be sent unless a one cent stamp, to prepay postage, accompanies the order.

A NEW YORK CREMATION SOCIETY.—This journal witnesses with satisfaction the endeavor being made in some of our larger cities to establish crematories and to familiarize the public with the neat, cleanly and wholesome method of disposing of the dead, by incineration. We are quite sure that cremation needs only to be witnessed by an intelligent people to commend it to them. The ignorant and superstitious will doubtless cling to the barbaric custom of inhumation, until their prejudice is removed by the example of the intelligent and refined element of communities. We are made glad therefore because of New York's cremation society.

A FOOT BRIDGE.—The *Sunday Republican*, now extinct, advocated the construction of a covered walk over the Lake and Main Street bridges. A far better and more convenient arrangement would be the building of a foot bridge on the upper side of the Railroad bridge. Years ago this project was contemplated and the railroad authorities interviewed as to whether permission could be obtained to build such a structure on the abutments and piers of the railway bridge. Assent was at that time given, which doubtless would be renewed upon proper application. A foot bridge at this point would be of unspeakable convenience to the denizens of the Fifth Ward.

GENEROSITY.—"O, that's nothing, he can well afford it," was a remark made to us this morning when we had related how a certain person had given a large sum of money to a charitable purpose. The remark was a thoughtless, ill-natured one, and too often indulged in by the people generally. It is not those who are abundantly able, that habitually give, but, contrariwise, are the ones most loath to contribute of their abundance. Generally, it is by means of a long continued practice of economy and deprivation of luxuries, and sometimes even necessaries, that men become rich and possessed of abundant means. It becomes, therefore, extremely difficult for them to give of their hard-earned riches, for charity's sake. Such an act is entirely the opposite of a custom and training that has governed them for the many years of a successful business career. Never, therefore, seek to minify the really generous gift—no matter how small—of a rich man.

DR. A. J. INGERSOLL, of Corning has been delivering medico-religious discourses in our city for several sundays past. He proclaims and reiterates a very ancient system of cure, in that "all men are healed through faith in Christ." The Doctor is a very pleasant and agreeable old gentlemen, and since he gives no medicine, can do little or no harm. His followers believe he does them good; and, since a man must *feel* well to believe that he *is* well, perhaps the doctor's cures are as real as though performed in the "regular" manner. We still adhere to the proposition made in a recent number of this journal, that every one should be permitted to make an ignoble ass of himself in the selection of his medical adviser if he so desires. This is not intended to apply to this particular case however, as we hear Dr. Ingersoll well spoken of and as laying some claim to intelligence.

"DO WE OWE OUR PARENTS ANYTHING?"—This question was recently discussed in a lecture before the R. Y. M. C. A. of this city by Ausburn Towner. Major Towner talks quite as entertainingly as he writes, beside giving one a deal of information upon any topic which he undertakes to handle. He is a very pleasing lecturer and easily surpasses, in interest and instruction, any of the high-priced professional lecturers that we have been accustomed to listen to.

There exist in our own city an array of gentlemen capable of giving a very pleasing course of lectures that would be of real value to our citizens. Suppose the society for the Home for the Aged should advertise a course of lectures to be delivered by these very pleasing speakers: Mr. Beecher, Prof. Ford, Ausburn Towner, Dr. Gleason, Frank Hall and Prof. Steele. They could hardly be excelled in their several departments and could not fail of attracting good audiences.

---

CLEAN UP.—Our readers need only be reminded that their back yards and lanes need to be thoroughly policed before the increasing temperature of the spring months causes pestilence to arise from the decomposing vegetable and animal matter that has found lodgment there during the long winter months now passed. Upon the surface of the ground will be found all manner of garbage that will surely endanger your family to disease, if not now speedily removed. Clean up therefore. Rake and sweep your premises. Bury the bones, peelings and dish-washings well under ground. Examine cess-pools and drains to learn that they are free and not overflowing into your wells. See that no surface water finds a way into your water supply, that your cellar is thoroughly cleansed and whitewashed top, sides and bottom. It has been clearly demonstrated that diphtheria, typhoid fever and kindred diseases lurk and thrive where nastiness most abounds. Now is the time to attack their haunts with shovel, hoe, rake and lime. Go at it.

---

CORONER'S INQUESTS.—A recent contributor to the *Advertiser* of this city gives a graphic and exceedingly amusing description of a coroner's inquest held upon the body of a child, in a neighboring hamlet, well known to have died from natural causes. The ridiculousness of having a number of "experts" testify to the post mortem appearances of the body, given in strictly technial language, to a jury of countrymen who are supposed to determine therefrom what produced death in the case under consideration, was revealed in all its grotesqueness. After the testimony of the doctors had been delivered, the jury are said to have been unable to determine whether the child died from hydrophobia or a thunderbolt. From the massive words used, they were inclined to the latter opinion, notwithstanding the Coronor interpreted the doctors as surmising that the child *may* have died from "natural causes." There exists altogether too much of this coroner investigation business. Two-thirds of it is entirely unnecessary, and employed solely as a means of support to a class of so-called doctors who are unable to obtain a livelihood by honest methods. The evil can never be remedied until "the people" elect suitable men for the office of Coroner. They will speedily discover that young and inexperienced doctors, without a remunerative practice, are not always the best appointments that can be made for such a position.

---

KISSING.—It always alarms us to have a stranger kiss our child. There is great danger in it. It is by no means an uncommon occurrence that the surgeon is called upon to treat an innocent little child for a dreadful constitutional disease imparted to it through an unclean kiss. Syphilis is more prevalent than the laity suspect. It lingers upon the lips of many a young man—and old ones too. It can be inoculated upon the lips of your child. Therefore, permit no stranger to kiss your children. Another filthy and dangerous ceremony is that of kissing a book in courts of justice. Such books generally do duty for a long while and become black and filthy with the slobberings of a multitude of witnesses. That syphilitic persons have kissed the book and deposited their saliva there, can scarcely be doubted. That syphilis can be communicated to you in kissing the same book is equally true. Therefore, when you swear in court, take your own bible with you, and refuse the nasty one. It is not only dangerous, but absolutely filthy. No person of refinement would think to place such a thing to his lips uncompelled by law. The practice should be abandoned.

---

MICROSCOPY.—One of the liveliest and most enthusiastic societies for the advancement of this interesting and delightful study anywhere to be found, exists in our own city. It has not yet been running one year, but has over fifty members, and at least one dozen fine instruments. The meetings are held monthly, are largely attended, and the greatest enthusiasm prevails.

Comparatively few people are aware of the beauties of nature that are hidden away in her secret recesses, where the most wonderful of her operations are performed in the dark wholly unobserved by the unaided eye. In these days, when every intelligent person is supposed to know something of the theory of "evolution," in which the very lowest forms of life are taken as a basis upon which to found a wonderfully interesting and fascinating theory of life, it becomes us to know something, for certain, of these minute creatures from which those seen by the naked eye, are supposed to have been evolved. One might read of a *monad*, an eternity without having the slightest conception of that minute, living cell, until he had seen him wiggle and twist under a good objective. An *amœba* might be expatiated upon, his psudopodia described, the marvelous manner in which he moves himself from place to place, dwelt upon and his singular manner of "feeding," talked about until dooms day, without getting the slightest idea of the reality until this curious gob of living protoplasm is shown you under the microscope. The beautiful creatures in the water —the infusoria, unique in form, marvelous in action; the exquisite algæ—the microscopic plants, than which nothing more beautiful is ever encountered in your conservatories, will delight you with new discoveries, in a single drop of water, for hours; the diatoms—those marvelously small and finely marked shells, handsomer by far than any found in your rambles upon the sea beach, will electrify you with their charming colors and delicate markings. Not only is the microscope an instrument for amusement, but also of the utmost importance to the physician in the study of diseased germs, the examination of secretions and tissues suspected to be concerned in certain ailments of the body, and in other ways made useful in his profession. A great impetus has been given microscopic research by reason of the very excellent instruments that have been perfected within two years, giving rise to numerous societies of gentlemen and ladies who, together, follow this interesting branch of investigation.

---

A CAT-ASTROPHY.—A cat was needed, imperatively demanded, for scientific purposes which we will not stop to define. This cat was to be injected with carmine—every capillary to be distended with that brilliant fluid while being divested of its own. The cat was caught, arrested in fact while comfortably snoozing under the kitchen stove. He was a homely, "singed cat," which circumstance lends some comfort to the narration which follows. He was transported to the Laboratory and preparations made for the administration of chloroform. An attempt was made to thrust him in a large box, which manœuver he resisted with all his might, his back humping, his tail swelling, and his claws clinching everything with which he came in contact, until he assumed the size angles and scratching capabilities of a dozen cats,—a dry goods box was not large enough to contain him. It is a perfectly wonderful how a cat can fasten his claws into anything absolutely out of his reach. His fore legs seem to be built on the Japanese-fishing-rod plan, so that he can blow them out to any desired length. We are satisfied that cat could not be placed in the middle of a box twelve feet square, without his catching on the edge; nor do we believe he could be dropped from a balloon, into a twelve acre lot without those everlasting claws of his fastening into the fence. Inasmuch as no box could be found into which he would fit, one of the boys volunteered to hold him while the other administered the chloroform. As the sponge approached his nose, he smiled naively, then grinned, blinked his eyes, while his tail moved restlessly from side to side and swelled to enormous proportions. Suddenly, without the slightest warning, his back appeared in a fearful crook, when, with an explosive sort of a sis-sis-spat, and a wild, unearthly wail of despair, he bounded onto the very top shelf of the laboratory from whence he dislodged acids, alkalies and various solutions into the beaker glasses and retorts below. And there he persisted in remaining, his two eyes glowing like balls of fire, his ears drawn close to his head, his back arched like that of a dromedary and his tail resembling the old fashioned boa that in ancient times adorned the ladies' necks. And there we pinned him to the wall, by means of a forked stick, and held a sponge tied to a broom handle, and saturated with chloroform to his snorting nostrils, when we soon had the satisfaction of seeing him gracefully succumb to the inevitable and to the dissecting table. Beautifully colored sections of his tongue, (with trichina in it) liver, kidney, retina, brain, and divers other portions of his resistless frame now delight us under the microscope; but, it is a question whether they are really worth the twenty-five dollars it cost for repairs, in the laboratory, of the damage that blamed cat caused in resisting our well directed endeavor of giving him chloroform.

---

SARA'S ART GALLERY.—A recent trip to Philadelphia, during Sara Bernhardt's season in that city, afforded us an opportunity to inspect her works of art. Accompanied by a friend, himself an artist, and therefore fully qualified to aid us in making up an estimate of Sara's handiwork, we presented ourselves at the door of an art gallery, paid our twenty-five cents each as an admission fee, and proceeded to inspect the "works of art."

There were three large paintings, eleven small ones, and seven small India ink sketches. In sculpture, two bronze heads, and four pieces in marble.

Well, they were queer. Not without merit, some of them, but as a collection of paintings and sculptures to see which an admission fee is asked, they will not pass muster. Sara doubtless has talent for painting and modelling, and, could she make it a specialty, instead of a sort of recreation, might succeed fairly. In her paintings, she exhibits her love for the ghastly in art as she does in her acting. This is evinced in her largest picture, the "Young Girl and Death." Her best painting, and which has more of a finished appearance, is that of some macaws, the drawing and coloring of which manifest decided talent. Most of the paintings and India ink sketches were of herself, displaying her own comely figure in various scenes from her plays. They manifested more satisfaction with her own lovely person than skill as an artist. Her sculpturings possessed more merit than her paintings. A bronze head of some Frenchman was particularly good. A marble medallion of the head and shoulders of some sort of a nymph, supposed to be rising from the waves, was a very beautiful work, most admirably executed. If Sara really did "sculp" this, without the aid of a professional,——well, she's an artist. Her paintings all had an unfinished appearance, as though the artist had dropped her brush expecting to return every minute and finish what had been fairly "stained in." Sara's side show attracted large numbers of people, and served the purpose intended by her—as an advertisement of her larger venture upon the stage, but it was hardly worth the twenty-five cents she asked for it.

---

ANOTHER EDISON HUMBUG.—That greatly over-rated, so-called scientist, Edison, is in the field with another clap-trap which he denominates the "Edison Electric Garter." His previous patent medicine dodge, which he styled "Edison's Polyform," (recommended to cure all manner of ills) left very little or nothing to be desired in the line of curatives, but the "electric garter" promises even more wonderful results. "It will develop the ankle and foot," says the advertisement. Goodness! Will it? What lady will invest in anything of *that* sort, we should like to know? See here, Mr. Edison, can't you make it diminish the foot and ankle by wearing it upside down? "It adds marvelous grace and elasticity to the step." Then, Bridget, our cook, shall have one that she may glide like a sprite over our wooden floors, upon our headache days. "It gives great ease and comfort in walking, skating and rowing." No? does it though? Send one to Mr. Courtney, by all means. His legs, arms, and spinal column should glitter with them. To enumerate all the great benefits to be derived from this marvelous new invention of the somewhat famous electrician, would occupy more space than we can afford for a strictly gratuitous advertisement. We simply refer to it that we may make the comments following. Such an advertisement sufficiently demonstrates two conditions of affairs, both of which are lamentable: The ignorance of the general public of the laws of electricity, and the almost universally accepted but erroneous opinion that elictricity is life, or at least a great promoter of life and health. Even could one particle of electricity be generated in Mr. Edison's garter, not the slightest effect could be imparted to the wearer either for good or evil. Yet, thousands of people will be beguiled into buying such an absurdity simply because Mr. Edison's name is attached to it. Then, it is lamentable that such a man, for purposes of gain, should lend his name to a fraudulent contrivance, himself knowing it, if he knows anything, to be a downright swindle.

On a par with this is the "Electric Hair Brush," recommended to prevent hair falling out or turning gray. The virtue (?) in this brush consists in a magnetized steel rod, concealed under the back of the brush, whose power is indicated by means of a small compass that accompanies the brush. How electricity is communicated to the bristles and thence to the head of the numbskull that uses it, the contriver of the device does not stop to tell.

The "electric," disks or "batteries," worn by a ribbon about the neck, and other so-called "electric" contrivances, all depend upon the ignorance or thoughtlessness of the wearers. Such devices can only impart the same benefits that are obtained from carrying a "buck-eye" in your pocket to ward off rheumatism, or from nailing a horse shoe over your front door as a barrier to pestilence or witches. The two last mentioned "remedies" have the merit of being costless, and are in every respect equal to those sold under the several names given; and, for this reason, are greatly to be preferred.

